# Liver graft as a ‘Trojan horse’: manifestation of variegate porphyria in an 11-month-old girl with biliary atresia after living-related liver transplantation

**DOI:** 10.1093/gastro/goag020

**Published:** 2026-03-15

**Authors:** Ulrich Stölzel, Birgit Knoppke, Thomas Stauch, Maria I von Eichborn, Herbert L Bonkovsky, Michael Melter

**Affiliations:** Division of Internal Medicine II and Porphyria Center, Klinikum Chemnitz, D-09116 Chemnitz, Germany; University Children’s Hospital Regensburg (KUNO), University Hospital Regensburg, D-93053 Regensburg, Germany; Deutsches Kompetenz-Zentrum für Porphyriediagnostik und Konsultation, MVZ Labor Volkmann und Kollegen GbR, D-76131 Karlsruhe, Germany; Department of Dermatology, University Hospital Regensburg, D-93053 Regensburg, Germany; Section on Gastroenterology & Hepatology, Wake Forest University School of Medicine and Advocate Health Wake Forest Baptist Medicine, Winston-Salem, 27157 NC, United States; University Children’s Hospital Regensburg (KUNO), University Hospital Regensburg, D-93053 Regensburg, Germany

**Keywords:** biliary atresia, light sensitivity, liver transplantation, porphyria, variegate por, phyria

## Abstract

We report on an infant girl with biliary atresia, who, at the age of 6 months, received a living-related liver transplantation (LRLT), (segments II/III) from her 37-year-old healthy mother. Five months after LRLT, the child developed skin lesions on sunlight exposed skin areas. Based on plasma fluorescence scanning, biochemical findings and DNA testing variegate porphyria (VP) was diagnosed in the girl. In this remarkable case hepatic heme synthesis was induced in the transplanted liver through medication (metamizole), stress and infection (cholangitis), unmasking previously undiscovered partial enzyme deficiency of PPOX. LRLT with subsequent manifestation of heterozygous VP in very early childhood has not been described hitherto. Our report will increase awareness of “rare risks” for “rare diseases” in liver transplantation.

## Introduction

Variegate porphyria (VP) (OMIM: 176200) is caused by a deficiency of the seventh and penultimate enzyme in heme biosynthesis, namely protoporphyrinogen oxidase (PPOX) and may present with acute neurovisceral attacks [1]. Moreover, as the term ‘variegate’ implies photosensitivity with skin blisters and bullae can occur with or without neurovisceral symptoms. Or neurovisceral symptoms may be present with or without photosensitivity. The underlying *PPOX* gene mutations are autosomally dominantly inherited, with low phenotypic penetrance, <1% [2]. VP is classified as an acute hepatic porphyria because induction of the first enzyme of hepatic heme biosynthesis, delta-aminolevulinic acid synthase-1, is considered as ‘conditio sine qua non’ for the occurrence of skin symptoms as well as neurovisceral attacks [3].

Induction of delta-aminolevulinic acid synthase-1 is triggered by porphyrinogenic drugs, caloric deficiency, hormones, chronic alcohol consumption, infections and stress, etc. and reflected by elevated concentrations of porphyrin precursors delta-aminolevulinic acid (5-ALA) and porphobilinogen (PBG) in plasma and urine. Elevated 5-ALA and PBG, as well as accumulated porphyrins, such as fecal coproporphyrin isomer III and protoporphyrin IX, fluorescence emission scanning of neutral plasma, and DNA testing are diagnostic [4, 5]. 5-ALA is considered to be toxic in higher concentrations, causing neurovisceral symptoms. Photosensitivity is due to increased porphyrins in skin. With the exception of and in contrast to rare homozygous or compound heterozygous cases of PPOX deficiency, autosomal dominant VP rarely clinically manifests before puberty and usually does not present with developmental or language delay [6, 7]. For the first time, we report here on manifest autosomal dominant VP in an 11-month-old girl biliary atresia in whom the disease became clinically manifest following living-related liver transplantation (LRLT).

## Case report

A six-month-old girl with biliary atresia required and underwent LRLT (segments II/III) with donor biliary-recipient enteric anastomosis from her 37-year-old healthy mother. Immunosuppression after LRLT was with cyclosporine and prednisolone. The infant did not receive trimethoprim/sulfamethoxazole. Six weeks after LRLT, acute rejection (rejection activity index 5) was treated with prednisolone pulse therapy for 6 days. Eight weeks after LRLT, significant stenosis of the biliary-enteric anastomosis required percutaneous trans-hepatic cholangiography and dilatations. Recurrent cholangitis was treated with piperacillin, tazobactam, and meropenem.

**Figure 1:**
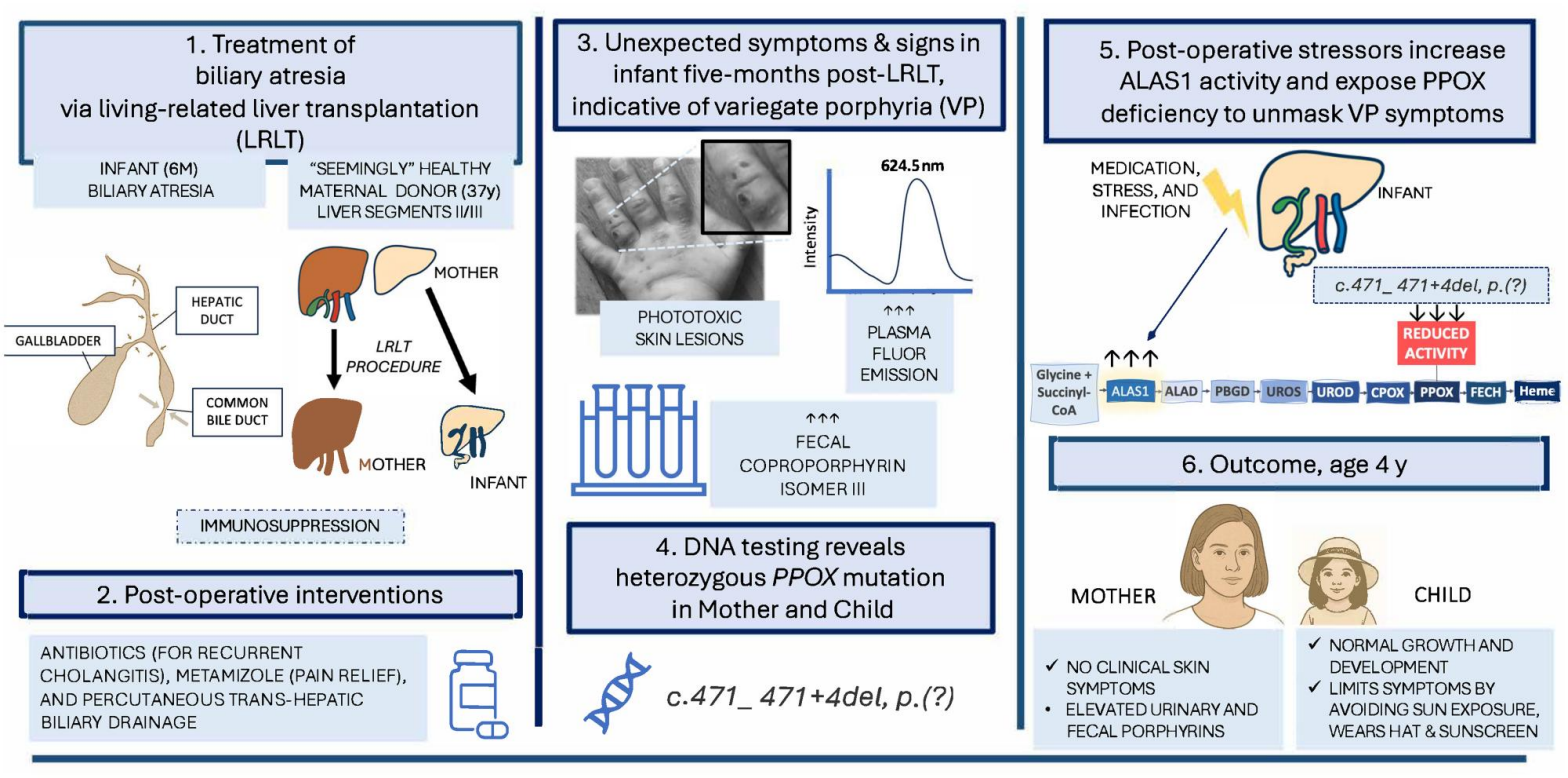
Summary of events before, during, and after LRLT for biliary atresia: the plasma fluorescence scan was performed on plasma obtained from the patient at 18 months of age, with an excitation wavelength of 410 nm in the patient aged 18-months, 12 months after LRLT from her mother. Emission maximum at 625.5 nm is unique and diagnostic for variegate porphyria. The images show fragile skin, wounds, crusty erosions, and blisters on light exposed hand. ALAS1 = delta-aminolevulinic acid synthase-1.

Five months after LRLT, the child developed very fragile skin on light-exposed areas (face and hands/fingers) (Fig. 1). Four  months later, she developed bullae, some of which broke and led to blisters that were slow to heal. Milia, hyperpigmentation, and scars developed. As a result, the child had a mixed appearance of her skin: blisters, wounds, crusty erosions, milia, hyperpigmentation, and scars. Among other causes, cutaneous porphyria was suspected. Based on plasma fluorescence scan with an emission maximum at 625.5 nm [excitation = 410 nm], and a high proportion of 90.9% fecal coproporphyrin isomer III (normal <35%), VP was diagnosed. Salient biochemical results are summarized in Table 1.

**Table 1:** Selected laboratory characteristics in an 18-month-old girl with biochemically and clinically manifest variegate porphyria 12-months after LRLT from her mother.^a^

	Urinary 5-ALA/Crea (mmol/mol)	Urinary PBG/Crea (mmol/mol)	Urinary URO/Crea (µmol/mol)	Urinary COPRO/Crea (µmol/mol)	Fecal porphyrins (µg/g)	Fecal COPRO-isomer III (%)	Peak plasma-Fluorescence (nm)^b^	URO plasma (µg/L)	COPRO Plasma (µg/L)	PROTO plasma (µg/L)
Child	7.34	0.63	5.12	23.59	12.2	90.9	625.5	11.3	5.8	9.2
Mother	0.22	0.14	3.44	28.5	121.1	80.4	626	–	–	–
Normal	<2.66	<1.01	<4.33	<20.68	<85	<35	Negative	<2	<4	<7

a At the time of these studies, the child displayed a cholestatic blood picture (serum gamma GT and alkaline phosphatase activities >10-fold and >2-fold upper limit of normal, respectively). The total serum bilirubin was normal.

b Excitation wavelength was 410 nm.

5-ALA = delta-aminolevulinic acid; Crea = creatinine; COPRO = coproporphyrin; PBG = porphobilinogen; PROTO = protoporphyrin; URO = uroporphyrin.

Subsequently performed sequencing of the *PPOX* gene revealed heterozygous *c.471_ 471 + 4del, p.(?)* variant in the girl, her mother, and the maternal grandfather. Her mother has done well before and after donating part of her liver with no skin lesions or other clinical features of VP. Urinary and fecal porphyrins are shown in Table 1.

The father of the girl has not been tested for *PPOX* mutations. The maternal grandfather did not report on any symptoms of acute porphyria, nor any history of cutaneous manifestations of VP.

Taken together, the skin lesions, plasma fluorescence screen results, high proportion of fecal coproporphyrin isomer III, and mutational findings establish the diagnosis of VP, which became clinically manifest only after LRLT, complicated by chronic cholestatic liver disease.

Currently, at the age of 4 years, infant growth and development are normal for Caucasian children. The weight is 16.5 kg (50th percentile for age), height is 102 cm (50th percentile for age), and her body mass index was 15.86 (50–75th percentile for age). The girl avoids sunlight whenever possible and always wears an oversized hat. Initially, for light protection on exposed skin, mineral-containing (zinc-, titanium oxide) sunscreens for children were used. Later on, in addition, tinted pigments containing sunscreens were favored. The skin’s sensitivity to light has decreased significantly. Blisters no longer appear. Only the skin fragility remains. Overall, in the wintertime, light sensitivity is less than in spring and summer.

## Discussion

We postulate that hepatic heme synthesis was induced in the transplanted liver through stress, medications, and immunological reactions, leading to an unmasking of previously clinically silent partial deficiency of PPOX. The ACMG classification of the *PPOX* variant *c.471_ 471 + 4del, p.(?)* variant is allocated as ‘likely pathogenic’ and so far, not recorded in the gnmoAD registry. Functional studies were not performed. Concerning the excretion values, it should be pointed out that the diagnostic criteria, which are often, but not always, applicable in adulthood, are less pronounced and often not textbook-like in childhood. In children, fecal porphyrin excretions are frequently much lower than in adults, and often we find concentration close to or even below the limit of quantification (LoQ). Therefore, in the described case, the proportions of the different porphyrin fractions were not determined. The mother showed a predominance of fecal coproporphyrin (coproporphyrin:protoporphyrin = 1.75:1) and an isomeric inversion with 80% isomer III (the latter being typical of VP). Several examples of adult, molecularly genetically confirmed VP have been observed in patients in whom coproporphyrin is dominant, harderoporphyrin or tricarboxyporphyrins appear, and only relatively comparatively similar amounts of fecal protoporphyrin are found (T. Stauch, unpublished results). Pre-analytical factors related to the low stability of protoporphyrin (microbial transformation or other degradation) may also explain the sometimes relatively low protoporphyrin fraction in VP. Biliary disorders after liver transplantation probably contributed to further accumulation of phototoxic porphyrins. Otherwise, liver stress caused by hepatitis A has also been reported to unmask PPOX deficiency and therefore VP [8].Notably, the mean age of the first phototoxic reaction in 55 patients with autosomal dominant VP from Finland was 26 years (range 14–56 years) [9].

Since sunscreens that have been used did not completely block the visible light, the improvement of skin symptoms may be considered to be coincidental. However, we feel that these sunscreens help to avoid light-induced skin damage. In general, all potential triggers as listed in the “Introduction” section were eliminated. Before diagnosing VP, during febrile episodes, the child received metamizole repeatedly. The latter substance is considered as porphyrinogenic and was not used after the diagnosis of VP had been established. Long-term surveillance, including porphyrin precursors 5-ALA, PBG, urinary and fecal porphyrins, liver and kidney function, is planned.

## Conclusion

This observation clearly shows the interplay among previously unknown reduced PPOX enzyme activity in the maternal liver graft and host-driven precipitating factors that led to clinically manifest autosomal dominant VP, even in early childhood. LRLT with subsequent manifestation of heterozygous VP in very early childhood has not been described hitherto. We hope that our report will increase awareness of ‘rare risks’ for ‘rare diseases’ in liver transplantation.

## Authors’ contributions

U.S. wrote the manuscript, collected information, and put forward the significance of the article. T.S. conducted laboratory analysis. U.S., B.K., and M.I.v.E. collected the data and cared for the patient. H.L.B. and M.M. revised the manuscript for important intellectual content. All authors read and approved the final version of the manuscript.
